# Downregulation of p70S6K Enhances Cell Sensitivity to Rapamycin in Esophageal Squamous Cell Carcinoma

**DOI:** 10.1155/2016/7828916

**Published:** 2016-08-09

**Authors:** Zhaoming Lu, Kezheng Peng, Ning Wang, Hong-Min Liu, Guiqin Hou

**Affiliations:** ^1^School of Pharmaceutical Sciences, Zhengzhou University, 100 Kexue Avenue, Zhengzhou, Henan 450001, China; ^2^Institute of Medicine, Zhengzhou University, 40 Daxue Road, Zhengzhou, Henan 450052, China; ^3^New Drug Research and Development Centre of Zhengzhou University, 100 Kexue Avenue, Zhengzhou, Henan 450001, China

## Abstract

It has been demonstrated that mTOR/p70S6K pathway was abnormally activated in many cancers and rapamycin and its analogs can restrain tumor growth through inhibiting this pathway, but some tumors including esophageal squamous cell carcinoma (ESCC) appear to be insensitive to rapamycin in recent studies. In the present study, we explored the measures to improve the sensitivity of ESCC cells to rapamycin and identified the clinical significance of the expression of phosphorylated p70S6K (p-p70S6K). The results showed that, after downregulating the expression of p70S6K and p-p70S6K by p70S6K siRNA, the inhibitory effects of rapamycin on cell proliferation, cell cycle, and tumor growth were significantly enhanced* in vitro* and* in vivo*. Furthermore, p-p70S6K had strong positive expression in ESCC tissues and its expression was closely related to lymph node metastasis and the TNM staging. These results indicated that p-p70S6K may participate in the invasion and metastasis in the development of ESCC and downregulation of the expression of p-p70S6K could improve the sensitivity of cells to rapamycin in ESCC.

## 1. Introduction

Esophageal squamous cell carcinoma (ESCC) is one of the most common and aggressive malignant tumors in China [1]. Although therapeutic strategies have been improved, the prognosis of ESCC patients is still poor because of the clinicopathological characteristics of ESCC such as rapid clinical progress, lymph node metastasis, local recurrence, and resistance to chemotherapeutic drugs [2, 3], which impel us to further explore the molecular mechanism in carcinogenesis and progression and treatment strategy of ESCC.

The mammalian target of rapamycin (mTOR) is an evolutionarily conserved serine/threonine protein kinase and can be activated by insulin, nutrients, and growth factors [4–6]. mTOR kinase exists in two complexes: mTOR complex 1 (mTORC1) and mTOR complex 2 (mTORC2). mTORC1 consists of Raptor, Lst8, FKBP38, Deptor, PRAS40, and mTOR; mTORC2 complex is built up by Rictor, Lst8, Sin1, Deptor, Protor, and mTOR. In mammals, mTORC1 plays key roles in ribosome biogenesis and cap-dependent mRNA translation through p70S6K, which is a major downstream effector of mTORC1. The abnormal activation of mTOR pathway has been demonstrated in many cancers [6–8]; the activated mTORC1 promotes the phosphorylation of p70S6K (p-p70S6K) and the releasing of phosphorylated eukaryotic elongation initiation factor 4E (eIF4E) binding protein 1 (4EBP1) from eIF4E, which ultimately result in enhanced translation of genes that are required for cell growth. Accumulating evidences have demonstrated that mTOR and its downstream effectors such as p70S6K and 4EBP1 have central roles not only in cell growth but also in tumor invasion and metastasis [6–8]. Therefore, mTOR pathway has been recognized as an important and attractive therapeutic target for cancer therapy [9, 10]. It has been shown that the inhibitors of mTOR such as rapamycin (Rapa), temsirolimus (CCI-779), and everolimus (RAD001) can reduce tumor cell size and inhibit cell proliferation by inhibiting mTOR pathway, which have been studied both preclinically and clinically for treating a variety of tumor types [11–15]. But recently, it is increasingly recognized that rapamycin and its analogs (rapalogs) are not sufficient to achieve abroad and robust anticancer effects; some tumors are even not sensitive or have resistance to them [16–18]. To explore the methods to improve the sensitivity of ESCC cells to rapamycin, in the present study, the expression of p70S6K and p-p70S6K in EC9706 cells was downregulated by p70S6K siRNA; the changes of cell sensitivity to rapamycin in cell proliferation, cell cycle, and tumor growth were investigated* in vitro* and* in vivo*. Moreover, the expression and clinicopathological significance of p-p70S6K were analyzed in tissues of 35 ESCC patients. This study explores the method to improve the sensitivity of cells to rapamycin and provides a diagnosis target for ESCC patients.

## 2. Materials and Methods

### 2.1. Cell Culture, Animal Treatment, and Patient Tissue Samples

Human ESCC cell lines EC9706, ECa109, and EC1 were obtained from Type Culture Collection of the Chinese Academy of Sciences (Shanghai, China). Cells were cultured in RPMI/1640 medium with 10% FBS, 100 U/mL penicillin, and 100 *μ*g/mL streptomycin at 37°C in a humidified atmosphere consisting of 5% CO_2_, as described in our previous study [19]. Male athymic BALB/c nude mice (Slack King of Experimental Animals Co. Ltd., Wuhan, China) at 4 to 6 weeks of age and 18 to 22 g of weight were used in this study. All the animals were housed in independently ventilated cages (IVC) at a temperature of 25-26°C and a relative humidity of ~50%, lit 12 hours/day. All animal studies were carried out in compliance with the Guide for the Care and Use of Laboratory Animals of Henan Province, China. The data of patient tissue samples were described as in our previous paper [20].

### 2.2. Western Blot

ESCC cells were collected (or tissues of xenografts ground with liquid nitrogen in mortar) and lysed in protein lysis buffer and then centrifuged at 14,000 rpm for 15 minutes at 4°C and the supernatant was the total protein extracts. The protein concentration was determined by Bradford method [21]. Equivalent amounts of protein (30 *μ*g) were separated by SDS-PAGE and electrotransferred to a PVDF membrane by a semidry transferor. After incubated for 1 hour at RT in blocking buffer (5% skimmed milk in PBS-T containing 0.05% Tween 20), the membranes were incubated with indicated primary antibodies: anti-p70S6K, anti-p-p70S6K, and anti-GAPDH (Santa Cruz Biotechnology, USA) of 1 : 400 diluted in 2% skimmed milk in PBS-T, respectively, at 4°C overnight, followed by incubating with the appropriate HRP-conjugated secondary antibodies of 1 : 8000. Finally, the bands of specific proteins on the membranes were visualized with chemiluminescent substrate (Santa Cruz Biotechnology, USA) according to manufacturer's instructions. Between the incubations of every step described above, the membranes were rinsed three times with PBS-T.

### 2.3. Semiquantitative RT-PCR

After EC9706 cells transfected with p70S6K siRNA or negative control siRNA (Santa Cruz Biotechnology, USA) were cultured for different time, the expression of p70S6K mRNA was detected by RT-PCR. In brief, total RNA of EC9706 cells was extracted with Trizol reagent (Invitrogen, Carlsbad, USA) and reversely transcribed to cDNA using AMV First Strand DNA Synthesis Kit (Biotech Company, Shanghai, China). The PCR amplification mixture (25 *μ*L) consisted of 0.5 *μ*L cDNA mixture, 0.5 U Taq DNA polymerase, 2.5 *μ*L 10x PCR buffer, 2.5 mM dNTP mixture, and 50 pM of each of sense and antisense primers. The sequence of primers for p70S6K (204 bp) is forward primer 5′-ATG CTG CTT CTC GTC TGG-3′ and reverse primer 5′-TTG AGT CAT CTG GGC TGT-3′ and for GAPDH (internal control, 570 bp) is forward primer 5′-CAA GGT CAT CCA TGA CAA CTT TG-3′ and reverse primer 5′-GTC CAC CAC CCT GTT GCT GTA G-3′. The PCR conditions were as described previously [19]. The amplified products were subjected to electrophoresis on 1% agarose gel containing 0.2 *μ*g/*μ*L ethidium bromide and visualized under UV light.

### 2.4. Cell Proliferation and Cell Cycle Phase Analysis

Cell proliferation was determined by Cell Counting Kit-8 (CCK-8, Beyotime Institute of Biotechnology, China) according to manufacturer's instructions. Briefly, EC9706 cells transfected with p70S6K siRNA or negative control siRNA for 24 hours were harvested and seeded in a 96-well flat-bottomed plate (5 × 10^3^ cells/well) and cultured at 37°C for 24 hours. Subsequently, these cells were treated with rapamycin (Sigma Aldrich, USA) at different concentrations for 48 h. After 10 *μ*L CCK-8 reagent was added to each well, cells were incubated at 37°C for 4 hours and the absorbance was finally determined at 450 nm using a microplate reader (Bio-Rad Laboratories, USA). Each treatment group was assayed in triplicate for each group.

Cell cycle phase analysis was conducted by flow cytometry. Briefly, cells transfected with p70S6K siRNA for 24 hours were treated with 100 nM rapamycin for 48 hours. Cells were harvested and fixed in 70% cold ethanol and kept at 4°C. After cells were further incubated with RNase (50 *μ*g/mL) for 30 minutes at 37°C, 5 *μ*L propidium iodide (50 *μ*g/mL) was added to cell suspension and continued to incubate at RT for 30 minutes in the dark before analysis, and then the cell cycle phase was analyzed by flow cytometry. Cells with negative control siRNA were used as controls [22].

### 2.5. Xenograft Studies

Twenty athymic mice were divided into two groups of 10 mice each and were subcutaneously inoculated with EC9706 cells transfected with p70S6K siRNA or negative control siRNA for 24 hours, respectively. Briefly, cells with p70S6K siRNA or control siRNA were harvested, washed, and resuspended in PBS at 2 × 10^7^ cells/mL. A cell resuspension of 200 *μ*L (4 × 10^6^ cells) was inoculated s.c. into the left flank of athymic mice. For tumor growth analysis, the tumor size was measured every other day with a sliding caliper, and the tumor volume was defined as (longest diameter) × (shortest diameter)^2^/2. Further, tumor-bearing animals of the two groups were randomly subdivided into 2 subgroups of 5 animals each, respectively, and drug treatment was initiated when tumor volume reached 60–100 mm^3^ [23]. The treatment schedule was that the groups with p70S6K siRNA or negative control siRNA were injected i.p. with rapamycin (50 mg/kg) or PBS as controls, respectively, every other day for two and a half weeks. After the treatment was over, tumor-bearing mice were sacrificed and the tumors were removed, weighted, and then stored in liquid nitrogen till for protein analysis. All procedures were conducted in a laminar-flow biosafety hood. Inhibition rate = [(tumor volume of control group − tumor volume of experimental group)/tumor volume of control group] × 100%.

### 2.6. Immunohistochemical Analysis

The expressions of p-p70S6K in 35 tissues were measured by immunohistochemistry and the protocols in detail were described as in our previous study [20]. The anti-p-p70S6K antibody was used at a dilution of 1 : 200 and the evaluation of immunohistochemical results was performed by a pathologist without knowledge of the clinical and pathologic characteristics of these patients. The tumor cells in slides were scored according to the intensity (*I*), distribution (*D*), and pattern (*P*) reported by Dong et al. [24]: *I* score: 0, negative; 1, weak; 2, moderate; and 3, strong; *D* score (%): 0, negative; 1, 10–50%; 2, 51–90%; and 3, >90%; *P* score: 0, no staining; 1, sporadic positive staining; 2, focal positive staining; and 3, diffuse positive staining. The total scores of each tissue = *I* × *D* × *P*, and the 0 score was negative and ≥1 score was positive. The relationship between the expression levels of p-p70S6K and differentiation degree, depth of infiltration, lymph node metastasis and TNM stage, and the expression relevance of p-p70S6K with mTOR (data of mTOR expression has been reported in our previous study [19]) were analyzed, respectively.

### 2.7. Statistical Analysis

The results of all experiments were analyzed by standard Chi-square test or one-way analysis of variance where it was appropriate, using SPSS 16.0 (SPSS, Chicago, USA). All summary statistics were expressed as mean ± SD. In all statistical analyses, *P* < 0.05 was considered statistically significant.

### 2.8. Study Ethics Approval

The study was approved by the Ethics Committee of Zhengzhou University, Henan, China.

## 3. Results

### 3.1. Protein Expression of p70S6K/p-p70S6K and Effect of Rapamycin on Them in ESCC Cell Lines

**Figure 1:**
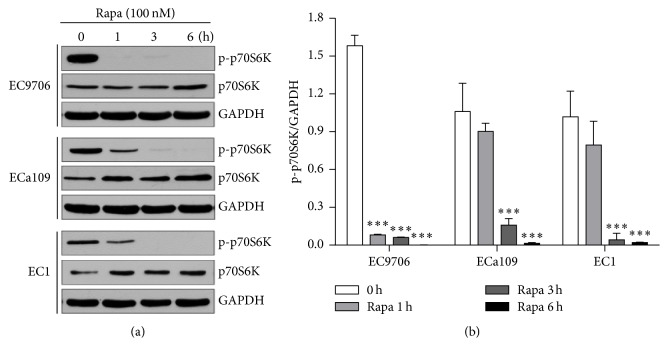
Effects of rapamycin on protein expressions of p70S6K/p-p70S6K in ESCC cell lines. (a) Antibodies to p-p70S6K and p70S6K, respectively. (b) Semiquantitative values from three independently repeated experiments, which were statistically analyzed by densitometry using software Image J (NIH, USA), are expressed as means ± SD.  ^*∗∗∗*^ *P* < 0.001 compared to untreated cells. GAPDH was used as loading control.

After EC9706, ECa109, and EC1 cells were exposed to 100 nM of rapamycin for different time (0, 1, 3, and 6 h), respectively, the protein expressions of p-p70S6K and p70S6K were analyzed by Western blot. The results showed that p-p70S6K had obvious expression in the three cell lines, which had the highest expression level in EC9706 cells. After cells were treated with rapamycin, the expression of p-p70S6K was obviously reduced in the three ESCC cell lines, especially in EC9706 cells, and rapamycin obviously inhibited the expression of p-p70S6K at a short time (1 hour) compared to that in the other two ESCC cell lines (*P* < 0.001, Figure 1).

### 3.2. p70S6K siRNA Downregulated the Expression of p70S6K mRNA and Protein in EC9706 Cells

**Figure 2:**
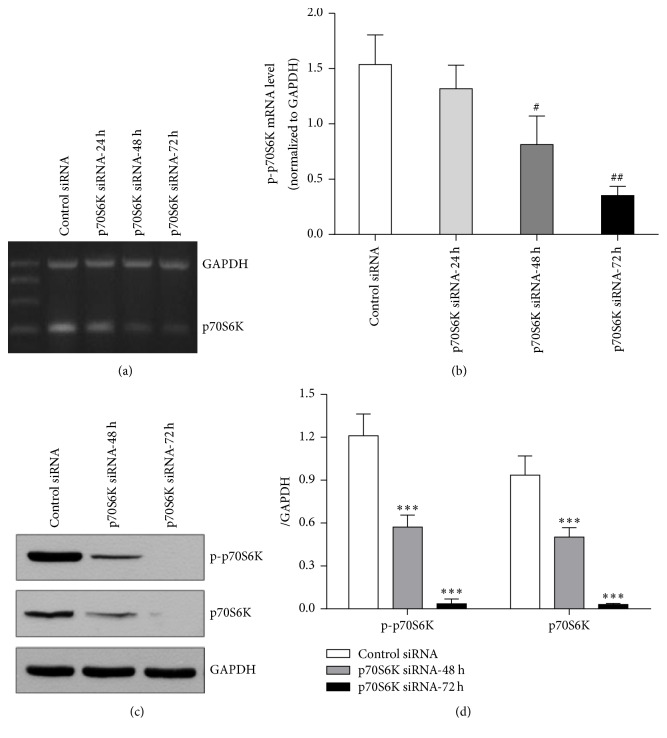
Interference efficiency of p70S6K siRNA on mRNA and protein of p70S6K in EC9706 cells. (a) Expression of p70S6K mRNA. (b) Semiquantitative values of p70S6K mRNA normalized to GAPDH mRNA. (c) Expressions of p70S6K/p-p70S6K protein. (d) Semiquantitative values of p-p70S6K/p70S6K protein normalized to GAPDH. Results were from three independently repeated experiments analyzed by using Image J (NIH, USA); data were expressed as mean ± SD. ^#^ *P* < 0.05, ^##^ *P* < 0.01 for mRNA results, and ^*∗∗∗*^ *P* < 0.001 for protein expression, compared to those of EC9706 cells treated with negative control siRNA.

To detect the interfering efficiency of p70S6K siRNA to the expression of p70S6K in EC9706 cells, the levels of p70S6K mRNA and protein in cells transfected with p70S6K siRNA for different times were measured by RT-PCR and Western blot. As shown in Figures 2(a) and 2(b), the expression levels of p70S6K mRNA decreased markedly in a time dependent manner. Compared to cells transfected with negative control siRNA, the inhibition rates of p70S6K siRNA on the expression of p70S6K mRNA were 14.76%, 43.75%, and 79.00% at 24, 48, and 72 hours, respectively. Moreover, the protein expression of p-p70S6K and p70S6K decreased significantly after cells were transfected with p70S6K siRNA and the decreasing ratios were 72.0% and 92.73% for p-p70S6K and 59.86% and 85.52% for p70S6K at 48 and 72 hours, respectively, compared to cells with negative control siRNA (Figures 2(c) and 2(d)). The results above showed that p70S6K siRNA could efficiently downregulate the expressions of p70S6K and p-p70S6K.

### 3.3. p70S6K siRNA Increased the Inhibition Effects of Rapamycin on Cell Proliferation and Cell Cycle of EC9706 Cells

**Figure 3:**
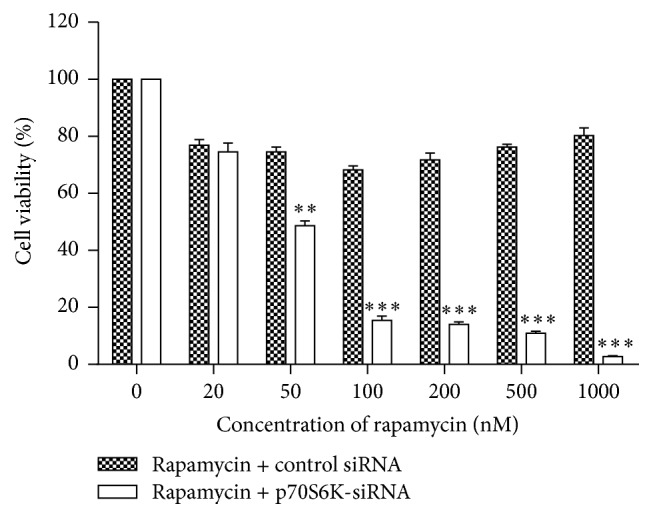
Effects of rapamycin on cell proliferation. Cells transfected with p70S6K siRNA or negative control siRNA for 24 hours were treated with rapamycin at different concentrations for 48 hours and cell proliferation was detected with CCK-8 kit. Data pooled from three independent experiments were expressed as mean ± SD. ^*∗∗*^ *P* < 0.01 and ^*∗∗∗*^ *P* < 0.001, compared to cells with negative control siRNA treated with rapamycin at the same concentration.

The results of cell proliferation showed that, in cells with negative control siRNA, rapamycin inhibited cell proliferation at low concentration (≤100 nM), while the inhibition effects receded along with the increase of rapamycin concentration (≥100 nM). And, compared to untreated cells, the inhibition rates were 23.20%, 25.47%, 31.73%, 28.26%, 23.77%, and 19.79% at 20, 50, 100, 200, 500, and 1000 nM of rapamycin, respectively. But after cells were transfected with p70S6K siRNA, the inhibition effects of rapamycin on cell proliferation were enhanced, and compared to untreated cells, the inhibition rates were 25.48%, 51.33%, 64.55%, 86.00%, 89.03%, and 97.30%, respectively. Furthermore, beginning with 50 nM, the inhibition effects of the same concentration of rapamycin on proliferation of cells with p70S6K siRNA increased obviously compared to that with control siRNA (*P* < 0.01 or *P* < 0.001, Figure 3). The results above showed that the inhibition effects of rapamycin on cell proliferation became strong after cells were transfected with p70S6K siRNA.

**Figure 4:**
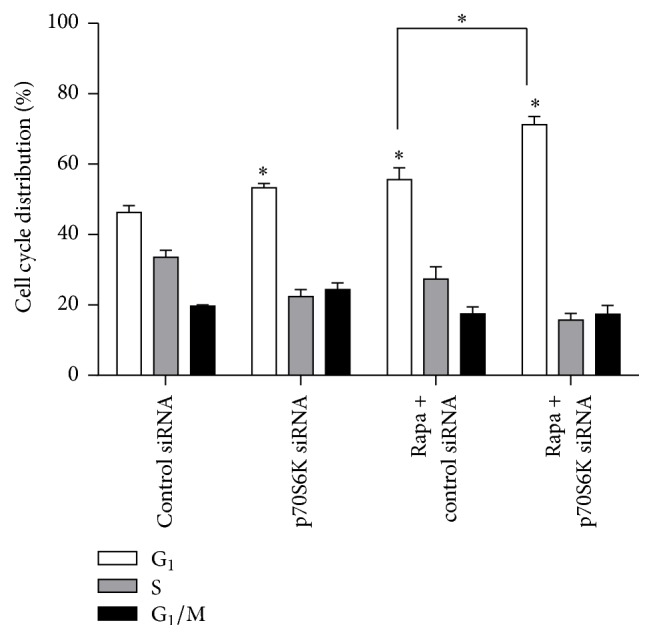
Effects of rapamycin on cell cycle phase. EC9706 cells were transfected with p70S6K siRNA or negative control siRNA for 24 hours and then treated with 100 nmol/L rapamycin for 48 hours. Cell cycle phase was analyzed by flow cytometry. ^*∗*^ *P* < 0.05, compared to cells with negative control siRNA.

The results of cell cycle analysis showed that rapamycin and p70S6K siRNA alone retarded cells to G_0_/G_1_ phase and the ratios of cells in G_0_/G_1_ phase were 57.87% and 53.82%, respectively, which had significant difference compared to control cells (ratio in G_0_/G_1_: 46.09%; *P* < 0.05). But when cells were treated with rapamycin combined with p70S6K siRNA, the ratio of cells in G_0_/G_1_ phase obviously increased and reached 73.73% (Figure 4). The results above indicate that p70S6K siRNA could promote the inhibition effects of rapamycin on the cell cycle phase of ESCC cells.

### 3.4. p70S6K siRNA Enhanced the Inhibition Effects of Rapamycin on Xenografts Growth of EC9706 Cells

**Figure 5:**
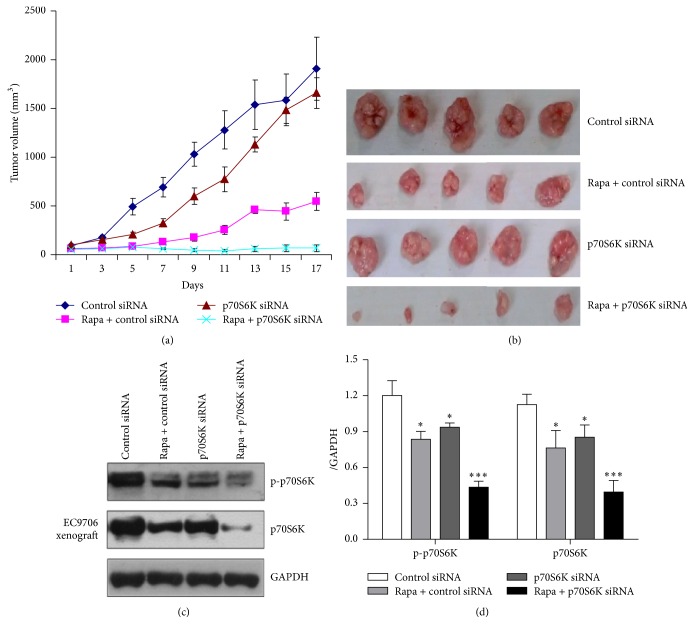
Tumor regression observed in EC9706 xenografts treated with different ways and the protein expressions of p70S6K and p-p70S6K in xenografts of each group. (a) Tumor volumes from the xenografts of each group were assessed every other day, and the results were expressed as means ± SE (mm^3^). The tumor growth of each treated group became slow, in which group treated with p70S6K siRNA combined with rapamycin was the slowest. (b) Tumors from the xenografts treated with different ways for two and a half weeks. (c) The protein expression of p70S6K and p-p70S6K in EC9706 xenografts analyzed by Western blot. (d) Semiquantitative values from three independently repeated experiments, which were statistically analyzed by densitometry using software Image J (NIH, USA), are expressed as means ± SD. ^*∗*^ *P* < 0.05, ^*∗∗∗*^ *P* < 0.001 compared to control group. GAPDH was used as loading control.

The effects of p70S6K siRNA on cell sensitivity to rapamycin* in vivo* were investigated by xenografts experiment. As seen from the curves of tumor growth (Figure 5(a)), the growth of tumors in every experimental group was slower than that in PBS group. p70S6K siRNA alone had a relative smaller effect on tumor growth (inhibition rate 13.06%; Table 1), rapamycin significantly inhibited the growth of xenografts, and the inhibition rate reached 70.35% (*P* < 0.05), while rapamycin combined with p70S6K siRNA had the strongest inhibitory effect (inhibition rate: 96.49%; *P* < 0.001), and the tumor growth nearly stopped (Figures 5(a) and 5(b)). The Western blot results in xenografts showed that the expression of p-p70S6K in rapamycin + p70S6K siRNA group was lower than that in rapamycin or p70S6K siRNA group (Figures 5(c) and 5(d)), which maybe explain the reason that p70S6K siRNA enhanced the inhibition effects of rapamycin on tumor growth* in vivo*.

### 3.5. Expression and Analysis of Clinical Significance of p-p70S6K in ESCC Tissues

**Figure 6:**
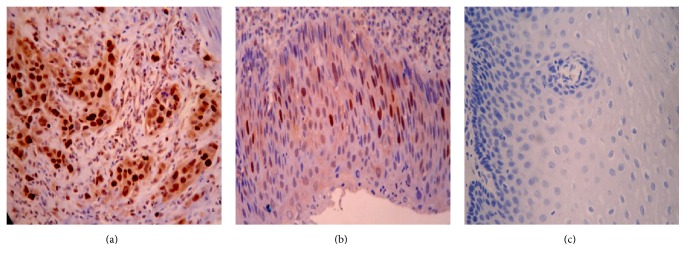
Expression of p-p70S6K in human normal esophageal and ESCC tissues by immunohistochemical analysis (×400). (a) Positive expression of p-p70S6K in ESCC tissues. (b) Moderate positive expression of p-p70S6K in dysplasia tissues of the esophagus (×400). (c) Negative expression of p-p70S6K in normal tissues of the esophagus (×400).

The immunohistochemistry results of p-p70S6K in 35 ESCC tissues showed that p-p70S6K was mainly expressed in the cell nucleolus (Figure 6). The positive expression rates of p-p70S6K were 33.3% (5/15), 73.3% (11/15), and 74.3% (26/35) in normal esophageal, dysplasia, and cancer tissues, respectively, which had a significant statistical difference among them (*P* < 0.05; Tables 2 and 3). The expression of p-p70S6K was not related to the histologic type and the depth of infiltration (both *P* > 0.05) but closely related to lymph node metastasis and the TNM stage (both *P* < 0.05). Furthermore, there were 19 tissues with positive expression of p-p70S6K in 22 tissues with positive expression of mTOR, while there were 6 tissues with negative expression of p-p70S6K in 13 tissues with negative expression of mTOR, indicating a positive correlation of the expression level between p-p70S6K and mTOR (*P* < 0.05; Table 4). The results above indicate that p-p70S6K might participate in metastasis and invasion of ESCC and could look as a diagnosis target of ESCC patient.

## 4. Discussion

Rapamycin, a macrolide antibiotic discovered from the bacterium* Streptomyces hygroscopicus*, was the first identified mTOR inhibitor and its anticancer effects were disclosed for the first time in 2002 [25], while rapamycin was not used in clinic but only in basic study about mTOR pathway because of its some drawbacks such as low aqueous solubility, poor oral bioavailability, and systemic toxicity [10]. CCI-779 and RAD001, the derivatives of rapamycin, have been proved by the FDA for treating some tumors such as advanced-stage renal cell carcinoma, pancreatic tumor, and hormone-receptor-positive advanced breast cancer [14, 15, 26], and RAD001 is also currently being tested as a single agent or in combination with additional therapies for the treatment of various cancer types [27]. However, although many papers have reported the antitumor effects of rapamycin and its derivatives in preclinical models of human tumors* in vitro* and* in vivo*, the efficacy of them as broad-based monotherapy for the treatment of cancer patients has not been as promising as initially expected because of their poor proapoptotic activity, not targeting all mTORC1 outputs, and the existence of multiple negative feedback regulatory loops [17, 26]. It has been shown that small interfering RNA (siRNA) and short hairpin RNA (shRNA) can effectively downregulate gene expression, modulating or selectively blocking the biological processes regulated by the target genes, and thus have been widely used in cancer research [28]. ESCC is a common cancer in China and had high mortality rate; we have conducted some previous studies about the mTOR pathway in ESCC and demonstrated the activation of mTOR pathway in ESCC [19, 20, 22]. Moreover, we found that some ESCC cell lines were not sensitive to rapamycin and even had resistance to rapamycin (paper in publishing by Disease of Esophagus). For exploring the method to improve the sensitivity of ESCC cells to rapalogs, we investigated the effects of rapamycin combined with RNA interference on ESCC* in vitro* and* in vivo* in the present study. Furthermore, the expression of p-p70S6K in tissues of clinical ESCC patients and clinic significance were analyzed for finding a diagnosis target of ESCC patients.

In this study, we showed that p-p70S6K had obvious expression and rapamycin suppressed the expression of p-p70S6K and promoted the expression of p70S6K in the three cell lines, while, in EC9706 cells, p-p70S6K had the highest expression and the inhibition effect of rapamycin on it was the strongest. Thus, we chose EC9706 cell line to explore the methods to improve the sensitivity of cells to rapamycin in the following experiments. Now that aberrant activation of mTOR/p70S6K pathway plays an important role in tumorigenesis and phosphorylated p70S6K by mTOR has higher activity to promote translation than p70S6K [6, 9], we speculated the combination of mTOR inhibitor and p70S6K siRNA could inhibit mTOR/p70S6K pathway at the most extent and thus inhibit tumor growth better. To verify our hypothesis, p70S6K siRNA was used in the present study for interfering with the expression of p70S6K, and then the changes of cell sensitivity to rapamycin were investigated in ESCC cells and xenografts. The results demonstrated that p70S6K siRNA downregulated the expression of p70S6K at mRNA and protein levels efficiently. When cells transfected with p70S6K siRNA were treated with rapamycin, the proportions of cells at G_0_/G_1_ phase increased significantly. Also, the inhibition effects of rapamycin on cell proliferation became strong and cells obtained again sensitivity to rapamycin of high concentration (>100 nM) after cells were transfected with p70S6K siRNA. The results above indicated that p70S6K siRNA improved the sensitivity of cells to rapamycin on cell proliferation and cell cycle* in vitro*. Moreover, the results of* in vivo* experiment further revealed that rapamycin had much stronger inhibition effects on the growth of tumors from cells transfected with p70S6K siRNA than that with control siRNA. Moreover, the Western blot results in xenografts tissues showed that the expressions of p70S6K and p-p70S6K were the lowest in rapamycin + p70S6K siRNA group, which might conform our speculation in molecular mechanism. Above all, inhibiting the expressions of p70S6K and p-p70S6K by siRNA remarkably improved the sensitivity of EC9706 cells to rapamycin both* in vitro* and* in vivo*.

To explore the roles of p-p70S6K, the key factor of mTOR pathway, in the progression of ESCC, we investigated the protein expression of p-p70S6K in ESCC tissues and analyzed its clinical significance and correlation with mTOR. Our results showed that p-p70S6K had a higher positive expression in ESCC tissues than that in atypical hyperplasia and normal esophageal mucosa tissues. Moreover, the expression of p-p70S6K was closely related to lymph node metastasis and the TNM stage of ESCC. We also confirmed that p-p70S6K had positive correlation with the expression of mTOR. Our results above suggest that both mTOR and p-p70S6K have higher expression in malignant type of ESCC tumors and may participate in the invasion and metastasis of ESCC, and p-p70S6K can be looked at as a target for evaluating malignancy grade of ESCC.

In conclusion, we propose that mTOR/p70S6K pathway has a central role in the progression and development of ESCC, and the expression of p-p70S6K would be of importance in clinical diagnosis of ESCC. In addition, based on our* in vitro* and* in vivo* results, rapamycin combined with p70S6K siRNA would be a suitable molecular therapeutic strategy for ESCC patients.

## Figures and Tables

**Table 1:** Effects of rapamycin alone or combined with p70S6K siRNA on the growth of ESCC xenografts in nude mice (*n* = 5).

Groups	Animal weight (g)	Volume before therapy (mm^3^)	Volume after therapy (mm^3^)	Inhibition rate (%)
Control	18.80 ± 1.92	82.68 ± 11.94	1907.86 ± 326.00	0
Rapamycin	20.07 ± 1.50	63.12 ± 23.18	546.62 ± 94.53	70.35^**∗**^
p70S6K siRNA	19.54 ± 0.63	87.99 ± 9.08	1658.72 ± 159.58	13.06
siRNA + Rapa	20.12 ± 1.28	61.14 ± 15.42	67.03 ± 33.88^**∗**^	96.49^**∗**^

^*∗*^ *P* < 0.05, compared with control group.

**Table 2:** Expressions of p-p70S6K protein in different tissues.

Tissue type	p-p70S6K				*P*
	*n*	−	+	Positive rate (%)	
Normal	15	10	5	33.3	0.015
Dysplasia	15	4	11	73.3	
Cancer	35	9	26	74.3	

**Table 3:** Clinical significance of p-p70S6K protein expression.

Pathological features	*n*	p-p70S6K	
		Positive *n* (%)	*P*
Histology classification			
I	9	7 (77.8)	0.942
II	14	10 (71.4)	
III	12	9 (75.0)	

Depth of infiltration			
Mucosa	7	4 (57.1)	0.404
Muscle layer	15	11 (73.3)	
Fiber membrane	13	11 (84.6)	

Lymph node metastasis			
No	19	11 (57.9)	0.016
Yes	16	15 (93.8)	

TNM phase			
I, II	13	7 (53.8)	0.033
III, IV	22	19 (86.4)	

**Table 4:** Correlation of the expression level of p-p70S6K and mTOR in ESCC tissues.

p-p70S6K	*n*	mTOR		*P*
		+	−	
+	26	19	7	0.034
−	9	3	6	
